# The Influence of Supplemental Dietary Linoleic Acid on Skeletal Muscle Contractile Function in a Rodent Model of Barth Syndrome

**DOI:** 10.3389/fphys.2021.731961

**Published:** 2021-08-19

**Authors:** Mario Elkes, Martin Andonovski, Daislyn Vidal, Madison Farago, Ryan Modafferi, Steven M. Claypool, Paul J. LeBlanc

**Affiliations:** ^1^Faculty of Applied Health Sciences, Center for Bone and Muscle Health, Brock University, St. Catharines, ON, Canada; ^2^Department of Physiology, Johns Hopkins University School of Medicine, Baltimore, MD, United States

**Keywords:** soleus, contractile kinetics, time to peak twitch, half-relaxation time, cardiolipin, tafazzin

## Abstract

Barth syndrome is a rare and incurable X-linked (male-specific) genetic disease that affects the protein tafazzin (Taz). Taz is an important enzyme responsible for synthesizing biologically relevant cardiolipin (for heart and skeletal muscle, cardiolipin rich in linoleic acid), a critical phospholipid of mitochondrial form and function. Mutations to Taz cause dysfunctional mitochondria, resulting in exercise intolerance due to skeletal muscle weakness. To date, there has been limited research on improving skeletal muscle function, with interventions focused on endurance and resistance exercise. Previous cell culture research has shown therapeutic potential for the addition of exogenous linoleic acid in improving Taz-deficient mitochondrial function but has not been examined *in vivo*. The purpose of this study was to examine the influence of supplemental dietary linoleic acid on skeletal muscle function in a rodent model of Barth syndrome, the inducible Taz knockdown (TazKD) mouse. One of the main findings was that TazKD soleus demonstrated an impaired contractile phenotype (slower force development and rates of relaxation) *in vitro* compared to their WT littermates. Interestingly, this impaired contractile phenotype seen *in vitro* did not translate to altered muscle function *in vivo* at the whole-body level. Also, supplemental linoleic acid attenuated, to some degree, *in vitro* impaired contractile phenotype in TazKD soleus, and these findings appear to be partially mediated by improvements in cardiolipin content and resulting mitochondrial supercomplex formation. Future research will further examine alternative mechanisms of dietary supplemental LA on improving skeletal muscle contractile dysfunction in TazKD mice.

## Introduction

Skeletal muscles play many important roles in the body. As a result, muscle weakness can limit mobility, impair balance, and make breathing difficult, impacting quality of life. Barth syndrome is an example of muscle weakness and, although rare, is a serious genetic disorder that primarily affects males (Barth et al., 1999). Historically, boys with Barth syndrome die of heart failure or infection. Improved diagnoses and treatment strategies aimed at cardiac and immune dysfunction (Zegallai and Hatch, 2021) have resulted in significantly improved survival rates (Clarke et al., 2013). However, skeletal muscle weakness (Bohnert et al., 2016; Bittel et al., 2018; Hornby et al., 2019) and exercise intolerance (Spencer et al., 2011; Bashir et al., 2017; Hornby et al., 2019) persist and worsen over time, impacting individuals’ physical function and independence (Mazar et al., 2019). Interventions directed at skeletal muscle weakness have demonstrated modest improvements in exercise tolerance with endurance exercise (Cade et al., 2016) and muscle strength with resistance exercise (Bittel et al., 2018). However, it is unknown if alternative treatment strategies could also/further improve skeletal muscle function in Barth syndrome.

The primary genetic defect characteristic of Barth syndrome is a mutation to the gene that encodes for tafazzin (Taz; Bione et al., 1996). Taz is a transacylase that remodels cardiolipin (CL; Xu et al., 2006), a phospholipid found predominately in the inner mitochondrial membrane with a fatty acyl chain composition made up of mostly linoleic acid (LA) in heart and skeletal muscle (Schlame, 2013). CL is important for mitochondrial structure (cristae folding, organization of membrane-associated proteins that are part of the electron transport chain), which in turn influences function (energy production; Claypool, 2009). As such, less than optimal functioning of Taz, as seen with Barth syndrome, results in reduced CL content and LA composition, resulting in dysfunctional mitochondria. Thus, treatment strategies aimed at improving CL content rich in LA may prove helpful in improving mitochondrial form and function and, in turn, skeletal muscle function in Barth syndrome patients.

Upon the discovery that impaired CL remodeling was one of the key driving forces behind the pathophysiology of Barth syndrome, it was logical to examine the role of supplemental LA as a potential therapy. To date, three studies have examined the influence of exogenous LA on Barth syndrome, examining cultured fibroblasts (Valianpour et al., 2003), cardiomyocytes (Wang et al., 2014), and lymphoblasts (Xu et al., 2016) from Barth syndrome patients. Incubation of cells with LA led to increases in LA-rich CL (Valianpour et al., 2003; Wang et al., 2014), reduced monolysocardiolipin (MLCL):CL ratio (Wang et al., 2014; Xu et al., 2016), and improved mitochondrial respiration and contractile function (Wang et al., 2014). Despite these positive findings in cell culture, to our knowledge, no studies have examined the influence of supplemental LA on CL content and composition, and skeletal muscle function at the level of the whole organism. The preclinical Taz knockdown (TazKD) mouse model has been extensively used in the literature because it closely recapitulates Barth syndrome pathologies seen in human patients (Ren et al., 2019), including skeletal muscle weakness (Acehan et al., 2011; Soustek et al., 2011) and exercise intolerance (Powers et al., 2013; Schafer et al., 2018; Goncalves et al., 2021). As such, this preclinical model may prove important in testing the possibility and efficacy of supplemental LA treatment.

Past literature has demonstrated that dietary intakes of lipids influence membrane fatty acid composition due to constant membrane remodeling (Ferreri et al., 2016). However, this relationship does not apply to all types of dietary lipids. Rodent skeletal muscle membranes appear relatively unresponsive to dietary saturated and monounsaturated lipids yet somewhat responsive to polyunsaturated fatty acids (PUFA), namely, n-3 PUFA as a percent of total PUFA or “PUFA balance” when fed a moderate dietary intake of lipids (25% of total energy intake) over 8weeks (Abbott et al., 2010). Specifically, skeletal muscle membranes were very responsive to diets with a low PUFA balance (n-3 PUFAs making up <10% of total dietary PUFAs; slope=0.98) and somewhat responsive to diets with a moderate to high PUFA balance (n-3 PUFAs making up >30% of total dietary PUFAs; slope=0.16; Abbott et al., 2010). It is also important to note that the low PUFA balance diets were high in LA (35–70% of total lipids) compared to the moderate to high PUFA balanced diets, where LA was less than 30% of total lipids (Abbott et al., 2010). Although this study examined the response of all membrane phospholipids indiscriminately, prior literature suggests that CL is responsive to changes in dietary lipids in liver (Feillet-Coudray et al., 2014), heart (Feillet-Coudray et al., 2014), and skeletal muscle (Fajardo et al., 2015). Here, we examined the influence of moderate dietary lipid intake (25% of total energy) with high LA (70% of total lipids) and low PUFA balance (<10%) on skeletal muscle CL content and composition, MLCL:CL ratio and skeletal muscle contractile function in a mouse model of Barth syndrome.

## Materials and Methods

### Animals and Diets

As approved by the animal utilization protocol, 16-10-01, a colony of TazKD mice generated from breeders originally purchased from Jackson Laboratories (stock number 014648), were housed at Brock University in the Comparative Biosciences Facility. TazKD was induced *in utero* and maintained post-natal by administering doxycycline (625mg/kg chow; Envigo TD.01306) as described previously (Acehan et al., 2011). All mice were allowed access to food and water *ad libitum* and were housed in an environmentally controlled room with a standard 12:12-h light–dark cycle. Mice offspring positive for the Taz small hairpin RNA transgene were identified by PCR using primers (forward: 5'-CCATGGAATTCGAACGCTGACGTC-3'; reverse: 5'-TATGGGCTATGAACTAATGACCC-3'). Non-transgenic littermates treated with the doxycycline containing diet were used as WT controls. Upon weaning, TazKD and WT littermates were fed a normal (CON) or high LA safflower oil (25% of total energy; Envigo TD.180388) diet (Table 1), both containing doxycycline, for a period of 8weeks (TazKD CON, TazKD LA, WT CON, and WT LA). Over the course of the 8-week dietary treatment, mice were weighed every other day. Following the 8-week dietary intervention, all mice underwent an *in vivo* measurement to assess grip strength (Bioseb, Chaville, France; Mandillo et al., 2008). 24h following the *in vivo* measurement, mice were euthanized *via* cervical dislocation while under isoflurane anesthetic. Soleus was extracted and either snap frozen in liquid nitrogen and stored at −80°C (lipid analyses, qPCR, and Western blotting) or processed immediately (*in vitro* muscle contraction). In a separate group of animals, following the same 8-week dietary intervention, animals were euthanized *via* cervical dislocation while under isoflurane anesthetic, and whole hindlimb tissue was extracted for subsarcolemmal mitochondria isolation. All animal procedures were approved by the Animal Care and Utilization Committee at Brock University (file #18-03-01) in compliance with the Canadian Council of Animal Care.

**Table 1:** Nutrient content of the diets.

Ingredient	Chow diet	LA diet
Protein (% kcal)	24	18
Carbohydrate (% kcal)	58	57
Sucrose	10	10
Lipid (% kcal)		
Soybean oil	18	–
Safflower oil	–	23
Flaxseed oil	–	2
Vitamin mix (g/kg)^1^	10	10
Mineral mix (g/kg)^2^	35	35

1 Vitamin mix contained A (15IU/g), D_3_ (1.5IU/g), E (110IU/kg), K_3_ (50mg/kg), thiamin (17mg/kg), riboflavin (15mg/kg), niacin (10mg/kg), B_6_ (18mg/kg), pantothenic acid (33mg/kg), B_12_ (0.08mg/kg), biotin (0.4mg/kg), folate (4mg/kg), and choline (1,200mg/kg)

2 Mineral mix contained calcium (1%), phosphorus (0.7%), sodium (0.2%), potassium (0.6%), chloride (0.4%), magnesium (0.2%), zinc (70mg/kg), manganese (100mg/kg), copper (15mg/kg), iodine (6mg/kg), iron (200mg/kg), and selenium (0.23mg/kg).

### *In vitro* Muscle Contractile Function

**Figure 1:**
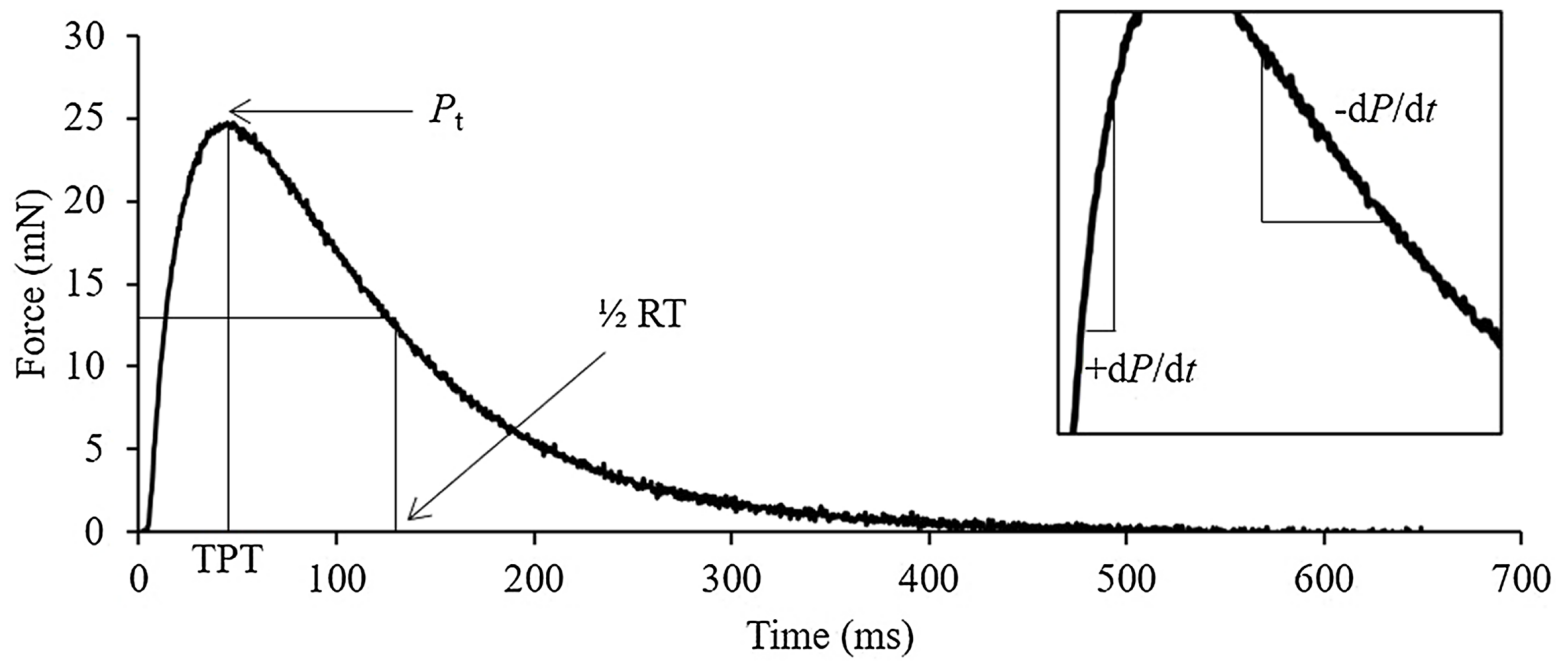
Representative soleus twitch trace identifying the key quantifiable measures of contraction. *P*_t_, peak twitch; TPT, time to peak tension; ½ RT, half-relaxation time; +d*P*/d*t*, rate of force development; and −d*P*/d*t*, rate of force relaxation.

Soleus from one hindlimb was dissected from distal to proximal tendon after being secured with braided silk sutures and was immediately mounted in an *in vitro* skeletal muscle testing system (Model 1200a; Aurora Scientific Inc.) for contractile measures as previously reported (Gittings et al., 2012; Mikhaeil et al., 2017). Data acquisition was performed using the Model 600a software, version 1.60 (Aurora Scientific Inc.) and muscle stimulation *via* flanking platinum electrodes driven by a Model 701B bi-phase stimulator (Aurora Scientific Inc.). Muscles were suspended in a jacketed organ bath-containing oxygenated (95% O_2_ and 5% CO_2_) Tyrode’s solution (121mm NaCl, 5mm KCl, 24mm NaHCO_3_, 0.4mm NaH_2_PO_4_, 0.5mm MgCl_2_, 1.8mm CaCl_2_, 5.5mm D-glucose, and 0.1mm EDTA, pH 7.4) maintained at 25°C (Lannergren et al., 2000), and left to equilibrate. To determine the optimal length (Lo) for maximal twitch force, muscles resting at just-taut length were stimulated every ~10s as muscle length was increased in small increments until peak active force was observed. Muscle length at Lo was determined using digital Vernier calipers. After preliminary procedures, the maximum isometric twitch and tetanic forces were obtained at Lo. Twitch force (*P*_t_) was measured as the maximum force produced in response to a single stimulation (i.e., 1Hz, 0.1msec pulse width; Figure 1), whereas peak tetanic force (*P*_o_) was determined by brief (500msec) supramaximal stimulation frequency (100Hz). Time to peak tension (TPT, the elapsed time from the onset to the peak of isometric force development) and half-relaxation time (½RT, elapsed time from the peak to 50% of isometric force development) of twitch force records were calculated. Rates of force development (+d*P*/d*t*) and relaxation (−d*P*/d*t*) were calculated as the maximal slope of the rise and fall in force during a twitch, respectively. Isolated muscles were also subjected to force-frequency (1–100Hz) and fatigue (70Hz for 350ms every 2s for 5min) protocols (Fajardo et al., 2016). Following the collection of contractile data, muscles were removed from the bath and the sutures were cut off immediately distal to the muscle–tendon junctions. Muscles were briefly blotted dry to remove excess liquid, and mass was determined on a standard mass balance. The calculation for physiological cross-sectional area was done as previously reported, using the optimal length and mass of each muscle (Grange et al., 2002).

### qPCR

RNA was isolated from frozen muscle using a Qiagen RNeasy Plus Kit (Qiagen, 74,136). cDNA was generated using EcoDry RNA to cDNA double-primed Reverse Transcriptase kit (Clontech, #639549). qPCR assays were performed on an ABI StepOnePlus Real-Time PCR System instrument (Applied Biosystems, #4376592) with KAPA SYBR FAST (Sigma, #KM4103) master mix and amplification efficiency-optimized primers (Integrated DNA Technologies, Coralville, IA). The primers used included Taz (forward 5' CCC TCC ATG TGA AGT GGC CAT TCC 3'; reverse 5' TGG TGG TTG GAG ACG GTG ATA AGG 3'; Acehan et al., 2011) and β-actin (forward 5' AAG AGC TAT GAG CTG CCT GA 3'; reverse 5' ACG GAT GTC AAC GTC ACA CT 3'; Huang et al., 2017). Threshold cycle (Ct) values were recorded and analyzed using the ΔΔCt method with expression of β-actin used as a reference gene.

### Lipid Analyses

Total lipids from muscle homogenates (1.25mg) were extracted (Folch et al., 1957), and CL content was analyzed by high-performance thin-layer chromatography (HPTLC) (Fajardo et al., 2017). MLCL and CL were separated by HPTCL on a separate plate (Lopalco et al., 2017), and MLCL:CL ratio was subsequently calculated using densitometry (ImageJ, Ver 1.53k, NIH, United States). LA composition of CL was analyzed by gas chromatography (Bradley et al., 2008; Fajardo et al., 2017).

### Western Blotting

Western blotting was performed to examine the protein expression of Taz as previously described (Fajardo et al., 2017) with the following modifications. Proteins from muscle homogenates (2μg) were solubilized using Laemmli buffer (Laemmli, 1970) and then electrophoretically separated using 12% standard glycine-based SDS-PAGE. Separated proteins were then transferred onto 0.2μm nitrocellulose membranes (Immuno-Blot, BioRad Inc., CA, United States) using a semi-dry transfer setting on the Trans-Blot Turbo Transfer System (BioRad Inc.). Membranes were immunoprobed with primary antibodies directed against Taz (Lu et al., 2016) diluted in 5% (w/v) milk in tris-buffered saline tween, then immunoprobed with a goat-anti mouse horseradish peroxidase conjugated secondary antibody (Jackson Immuno Research Labs, West Grove, PA), and detected with Clarity Western ECL Substrate with a BioRad Chemi Doc Imager (BioRad Inc.).

### Mitochondrial Supercomplex Analyses

Subsarcolemmal mitochondria were isolated from fresh whole hindlimb tissue as previously reported (Stefanyk et al., 2010). Blue-native polyacrylamide gel-electrophoresis was used to assess mitochondrial supercomplex formation as previously reported (Jha et al., 2016). Densitometry of supercomplexes from membranes was quantified (ImageJ, Ver 1.53k, NIH, United States) and expressed in reference to WT Con.

### Statistics

All values are expressed as the mean ± standard error (SE). All statistical analyses were performed using SPSS Statistics for Windows, version 25 (SPSS Inc., Chicago, Ill., United States). Two-way ANOVAs (genotype and diet) were performed on all data sets followed by a Tukey’s post-hoc analysis. For force-frequency curves, force was normalized to maximal force and a three-way mixed plot ANOVA was used to examine the main effects of frequency, genotype, diet, and their potential interactions. Frequency at 50% maximal force (F_50_) was calculated using the Hill equation. For fatigue curves, area-under-the-curve values were obtained for each muscle then averaged before conducting statistical comparisons. A value of *p*<0.05 was considered significant for all tests.

## Results

### Taz Transcription and Translation

**Figure 2:**
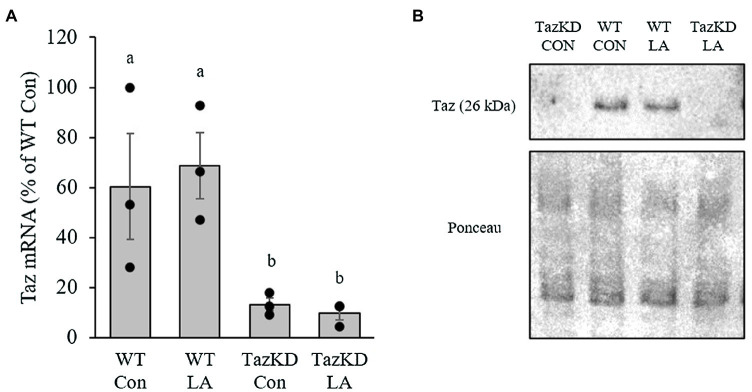
Administration of doxycycline in either control or supplemental linoleic acid diet resulted in a significant reduction in tafazzin (Taz; **A**) mRNA and **(B)** protein content in soleus of Taz knockdown (TazKD) male mice compared to their wild-type littermates. Values are means ± SEM, *n*=3 for Taz mRNA. WT, wild type; TazKD, Taz knockdown; Con, chow diet; and LA, linoleic acid-supplemented diet.

The presence of doxycycline in the diet (625mg/kg) for 8weeks significantly reduced Taz mRNA content in soleus of TazKD mice to ~10% of WT littermates (Figure 2A). This resulted in an absence of Taz protein in soleus of TazKD mice compared to WT littermates (Figure 2B). The presence of supplemental LA had no effect on Taz transcription and translation.

### Animal Morphometrics and *in vivo* Muscle Function

**Figure 3:**
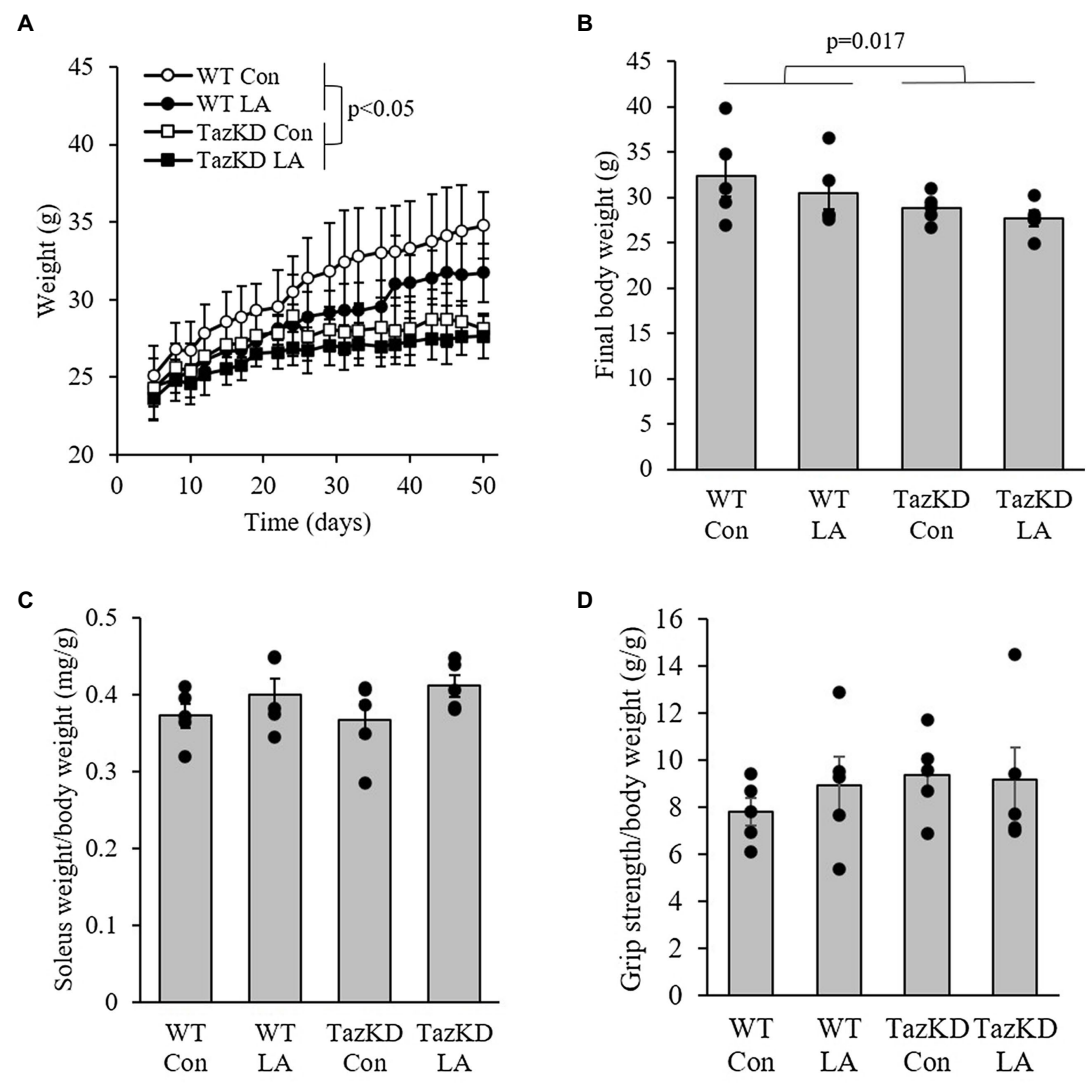
TazKD male mice were **(A)** slower growing and **(B)** had lower final body weight compared to their wild-type counterparts regardless of diet over the 8-week intervention. **(C)** Soleus weight as a fraction of body weight and **(D)** *in vivo* grip strength were not different between genotypes and diet. Values are means ± SEM, *n*=6. WT, wild type; TazKD, Taz knockdown; Con, chow diet; and LA, linoleic acid-supplemented diet.

TazKD mice were slower growing compared to WT littermates, regardless of diet (Figure 3A). This resulted in a significantly lower final body weight (Figure 3B). However, soleus weight as a fraction of body weight was not significantly different between genotypes or diet (Figure 3C). At the end of the dietary treatment, there was no difference between genotypes or diet in combined forelimb-hindlimb grip strength when controlled for body weight (Figure 3D).

### Cardiolipin Content and Composition and MLCL:CL Ratio

**Figure 4:**
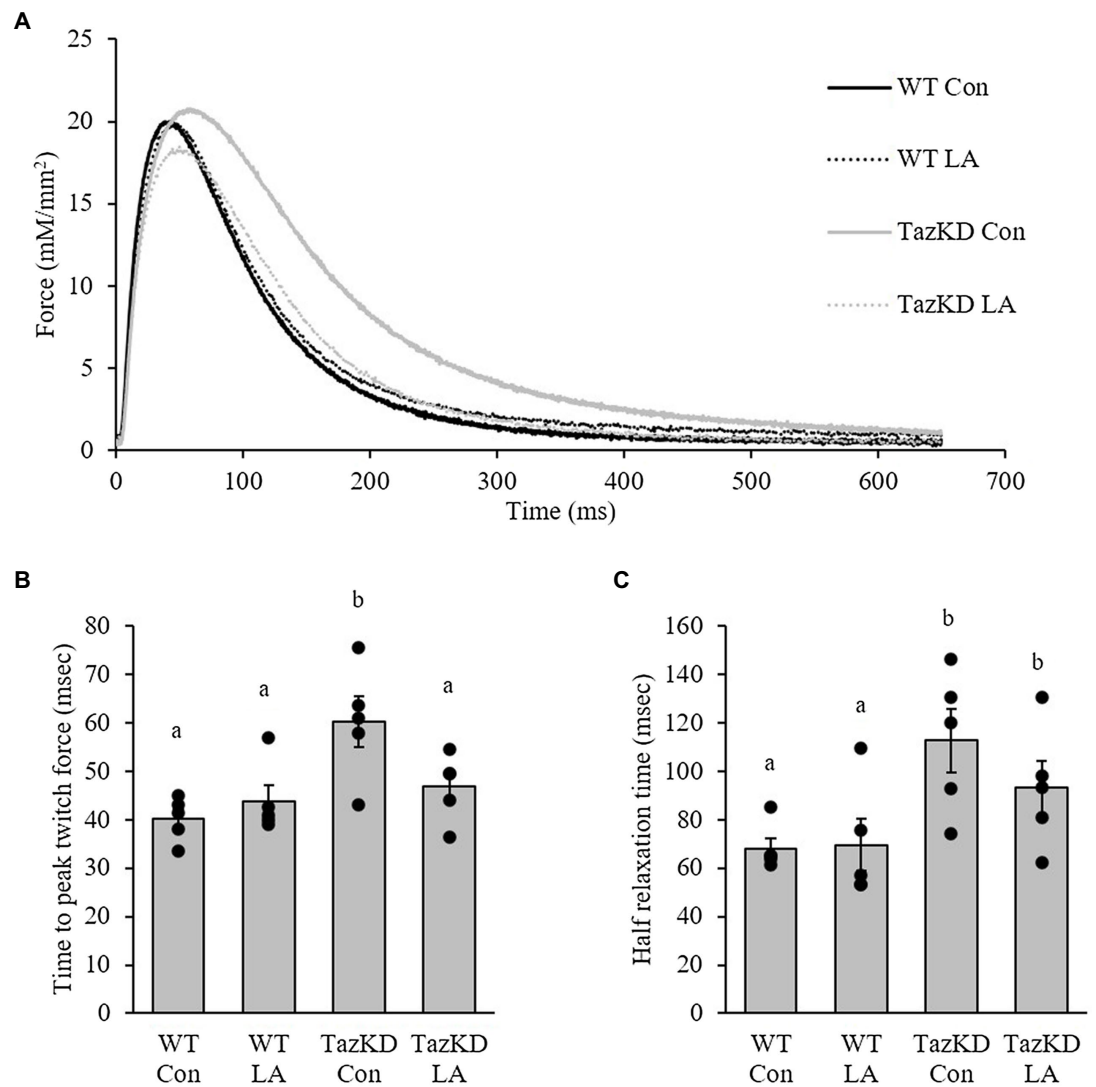
8 weeks of supplemental linoleic acid diet attenuated, but not significantly, the doxycycline-induced alterations in **(A)** cardiolipin content but had no influence on **(B)** cardiolipin linoleic acid content or **(C)** MLCL:CL ratio in soleus of TazKD male mice compared to their wild-type littermates. Values are means ± SEM, *n*=2–6; no statistical difference between values with the same letter (*p*<0.05). CL, cardiolipin; WT, wild type; TazKD, Taz knockdown; Con, chow diet; and LA, linoleic acid-supplemented diet.

CL content was significantly lower in TazKD soleus compared to WT, which was somewhat attenuated with supplemental LA in that TazKD was no longer significantly different from WT (Figure 4A). CL 18:2n6 composition was also lower in TazKD soleus compared to WT but was not affected by supplemental dietary LA (Figure 4B). MLCL:CL ratio was higher in TazKD soleus compared to WT and did not appear to be influenced by supplemental dietary LA (Figure 4C).

### *In vitro* Soleus Contractile Function

**Figure 5:**
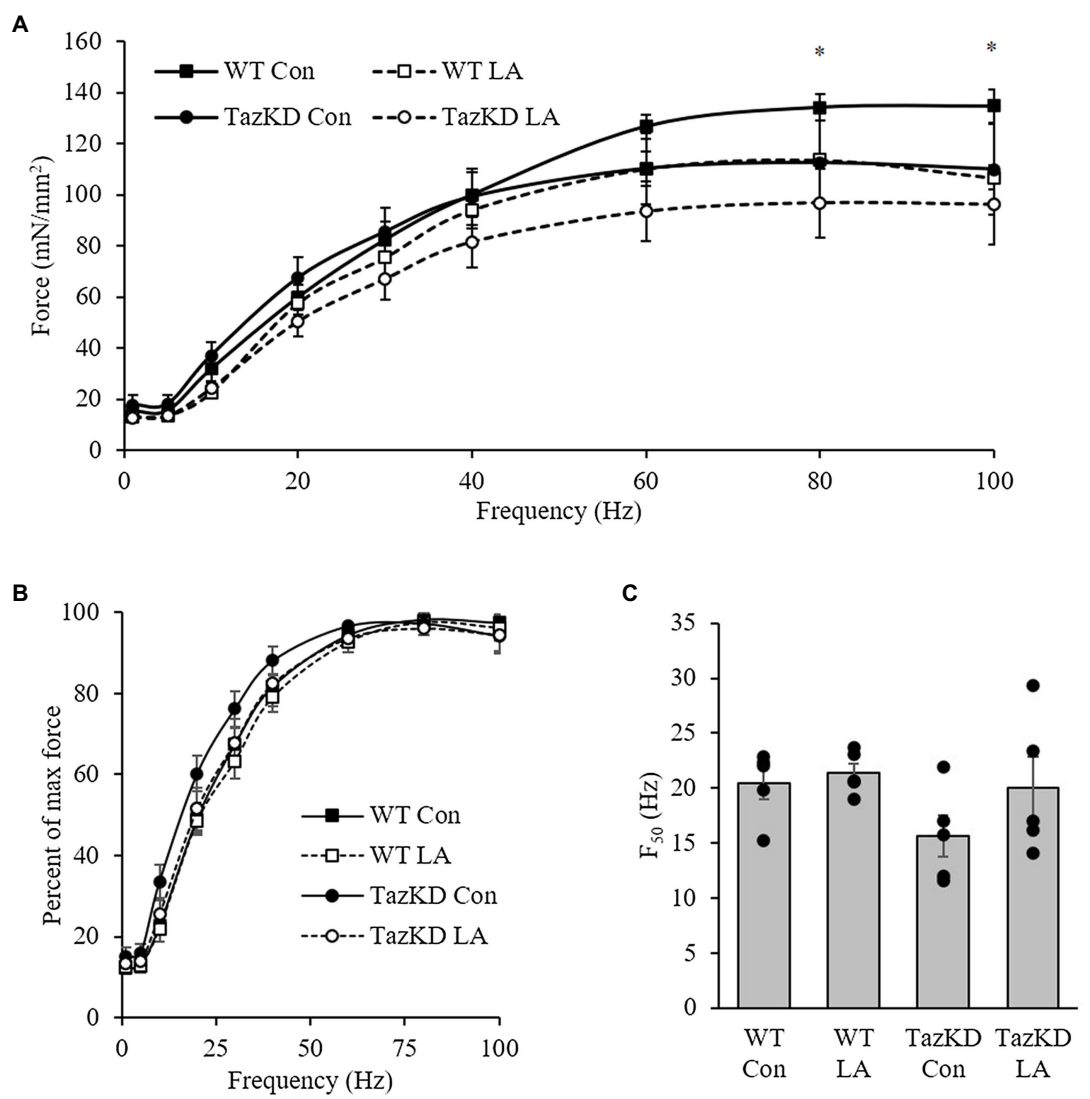
TazKD compared to wild-type male mice fed control diet show altered twitch kinetics as demonstrated by **(A)** the representative trace. **(B)** Time to peak twitch force was slower in TazKD soleus compared to WT and was attenuated by the LA diet. In comparison, **(C)** half-relaxation time was also slower in TazKD soleus compared to WT and was somewhat attenuated with the LA diet. Values are means ± SEM, *n*=5; no statistical difference between values with the same letter (*p*<0.05). WT, wild type; TazKD, Taz knockdown; Con, chow diet; and LA, linoleic acid-supplemented diet.

**Figure 6:**
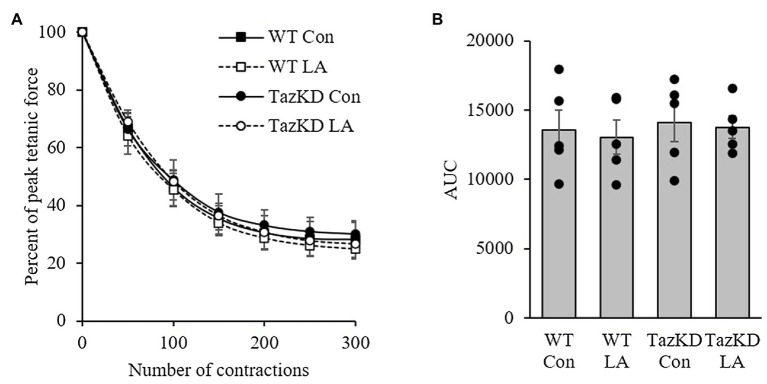
8 weeks of supplemental linoleic acid diet did not influence soleus fatigability expressed as **(A)** percent peak tetanic force and **(B)** area under the curve in TazKD male mice, or their wild-type littermates compared to the control chow diet. Values are means ± SEM, *n*=5. WT, wild type; TazKD, tafazzin knockdown; Con, chow diet; and LA, linoleic acid-supplemented diet.

**Figure 7:**
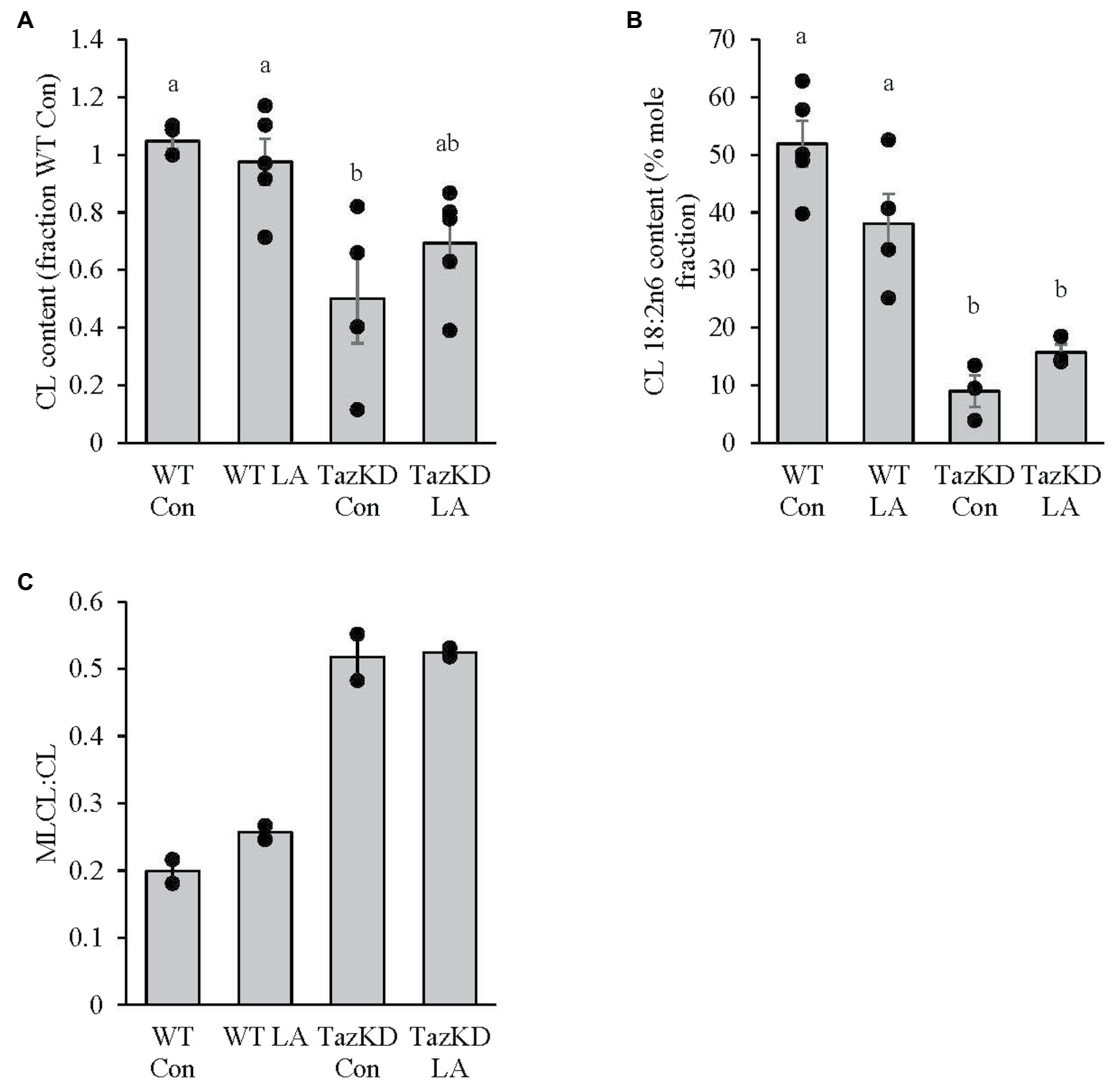
TazKD soleus demonstrated **(A)** lower absolute twitch force production at higher frequencies compared to WT but no significant differences between genotypes and diet when expressed as **(B)** a percent of max force or **(C)** the frequency at 50% of maximal force (F_50_). Values are means ± SEM, *n*=3–5. WT, wild type; TazKD, Taz knockdown; Con, chow diet; and LA, linoleic acid-supplemented diet.

Soleus from TazKD mice have impaired contractile properties compared to WT (Figure 5A), as demonstrated by a significantly slower TPT (Figure 5B) and ½ RT (Figure 5C). The addition of supplemental dietary LA significantly reversed the slow TPT in TazKD mice to levels similar to WT littermates. In terms of ½ RT, this was somewhat attenuated in TazKD with dietary supplemental LA but did not reach significance. Similar to previous research, absolute force production at higher frequencies was lower in TazKD compared to WT littermates but this was not impacted by dietary supplemental LA (Figure 6A) nor was it different between genotypes and diet when expressed as a percent of max force (Figure 6B) or the frequency at 50% of maximal force (Figure 6C). When examining peak twitch force, peak tetanic force, rates of force production, rates of force relaxation (Table 2), and rates of fatiguability (Figure 7), only rates of force relaxation showed a genotype-specific difference.

**Table 2:** Isometric muscle function in response to 8 weeks of control or supplemental linoleic acid diet in soleus of doxycycline-induced tafazzin knockdown mice and their wild-type littermates.

	WT		TazKD	
	Con	LA	Con	LA
*P*_t_ (mN)	20±2	20±4	21±5	18±2
*P*_t_/CSA (mN/mm^2^)	15±2	14±2	18±5	14±2
*P*_o_ (mN)	168±26	154±14	132±16	138±14
*P*_o_/CSA (mN/mm^2^)	127±23	110±15	116±19	106±13
+d*P*/d*t* (mN/msec)	1.0±0.1	0.8±0.1	0.8±0.2	0.8±0.1
−d*P*/d*t* (mN/msec)^*^	0.17±0.02	0.15±0.03	0.10±0.04	0.13±0.03
*P*_t_:*P*_o_	0.12±0.01	0.13±0.02	0.15±0.02	0.13±0.02

Values are means ± SEM, *n*=5; WT, wild-type; TazKD, tafazzin knockdown; Con, chow diet; LA, linoleic acid-supplemented diet; *P*_t_, peak twitch force; CSA, cross-sectional area; *P*_o_, peak tetanic force; +d*P*/d*t*, rate of force development; and −d*P*/d*t*, rate of force relaxation.

* Denotes main effect for genotype.

### Mitochondrial Supercomplex Formation

**Figure 8:**
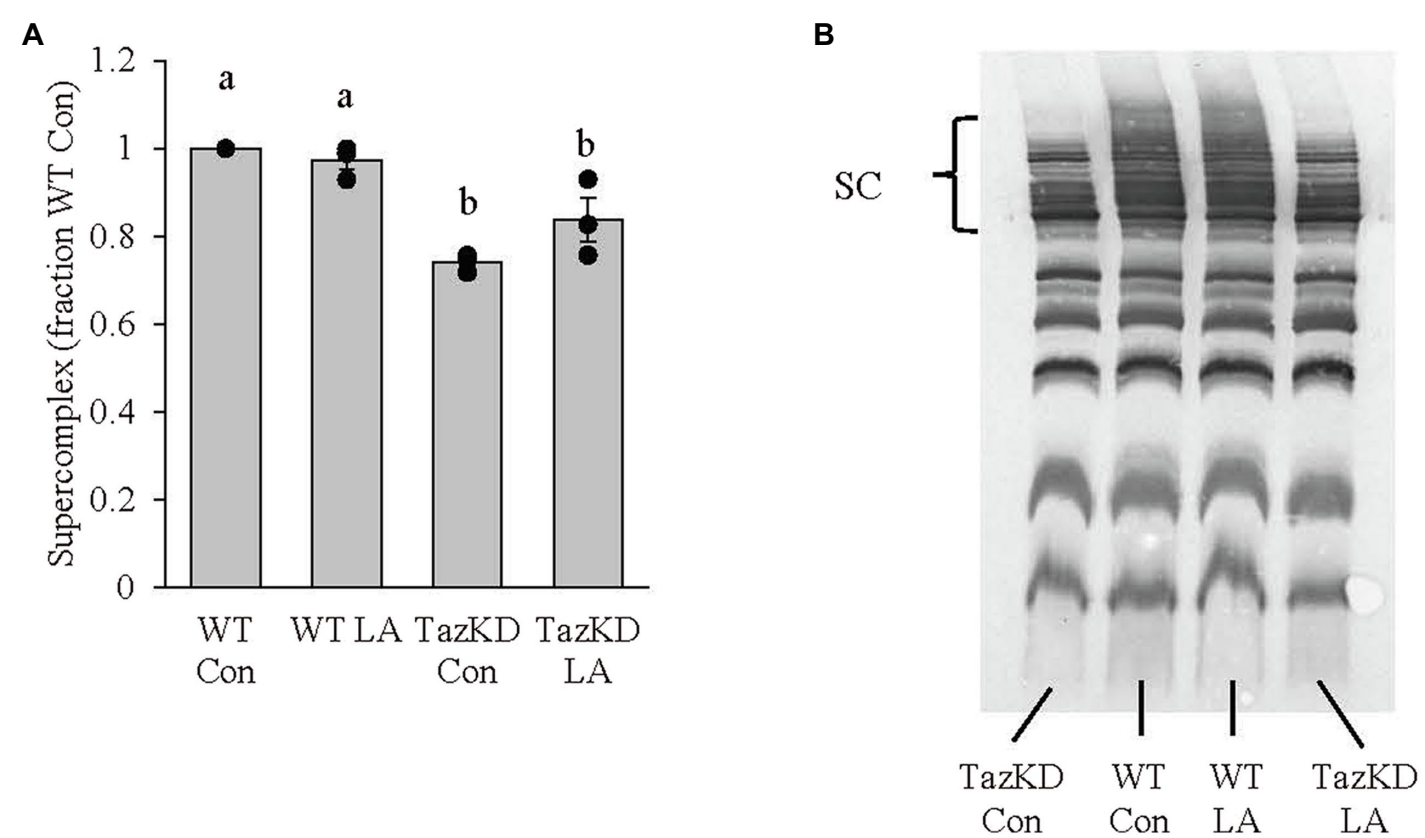
Subsarcolemmal mitochondria isolated from whole hindlimb of TazKD male mice demonstrated **(A)** lower supercomplex formation compared to wild-type littermates, which was not significantly increased (*p*=0.14) with 8weeks of supplemental linoleic acid diet. **(B)** Example membrane from blue-native polyacrylamide gel electrophoresis. Values are means ± SEM, *n*=3. WT, wild type; TazKD, tafazzin knockdown; Con, chow diet; LA, linoleic acid-supplemented diet; and SC, supercomplex.

Subsarcolemmal mitochondria isolated from whole hindlimb from TazKD mice had lower supercomplex formation compared to WT littermates (Figure 8). Although 8weeks of dietary supplemental LA somewhat attenuated the impaired supercomplex formation, the finding was not significant (*p*=0.14).

## Discussion

To our knowledge, this is the first study to fully characterize an impaired contractile phenotype in the pre-clinical rodent model of Barth syndrome and the potential efficacy of dietary supplemental LA to reverse this phenotype. Similar to previous studies, administration of doxycycline to TazKD mice *in utero* and post-weaning resulted in decreased skeletal muscle Taz mRNA, protein, CL content, CL 18:2n6 composition, and increased MLCL:CL ratio (Acehan et al., 2011; Soustek et al., 2011; Ikon et al., 2018; Johnson et al., 2018; Goncalves et al., 2021), along with reduced weight gain (Acehan et al., 2011; Cole et al., 2016; Ikon et al., 2018; Johnson et al., 2018; Goncalves et al., 2021). The main findings are (1) TazKD soleus demonstrated slower force development and relaxation *in vitro* as key contributors to this impaired contractile phenotype, (2) the impaired contractile phenotype seen *in vitro* in TazKD compared to WT did not translate to altered muscle function *in vivo*, and (3) supplemental LA attenuated, to some degree, soleus *in vitro* impaired contractile phenotype in TazKD mice, which appears to not be fully mediated by CL content and composition.

### TazKD Soleus Demonstrated Slower Force Development and Relaxation *in vitro* as Key Contributors to an Impaired Contractile Phenotype

This study represents the first to demonstrate altered skeletal muscle contractile kinetics *in vitro* in a mouse model of Barth syndrome. Soleus from TazKD male mice had slower TPT, ½ relaxation time (½RT), and rate of force relaxation (−d*P*/d*t*). Changes in skeletal muscle contractile kinetics can be influenced by muscle fiber-type composition. Soleus in mice is ~31% type I, 49% type IIA, 12% type IIX, and 3% type IIB (Augusto et al., 2004; Bloemberg and Quadrilatero, 2012) and demonstrates fiber-type shifting when challenged by unloading (Fajardo et al., 2017), endurance exercise (Kruger et al., 2013), lithium supplementation (Whitley et al., 2020), and aging (Bott et al., 2017) to name a few. To date, no research has examined the fiber-type composition of TazKD skeletal muscle. It is not likely that the observed changes in TPT, ½ RT, and −d*P*/d*t* in the current study would be due to changes in fiber-type composition as there were no differences in peak twitch force, peak tetanic force, or resistance to fatigue, skeletal muscle contractile variables that correlate with fiber-type composition (Stephenson et al., 1998). It is more likely that given Taz is an important enzyme to remodel CL necessary for proper mitochondrial function (Paradies et al., 2019), slower force development and relaxation in TazKD soleus may be related to the energetics of muscle contraction and relaxation, respectively.

One possible contributor to the slowed contractile kinetic phenotype seen in the current study is ATP availability. ATP, supplied by mitochondrial oxidative phosphorylation, is essential to fuel myosin ATPase for cross bridge cycling of myosin and actin during contraction and calcium reuptake into the sarcoplasmic reticulum by the sarco(endo)plasmic reticulum calcium ATPase (SERCA) during relaxation. Impaired mitochondrial supercomplex formation in TazKD compared to WT seen in the current study may limit ATP supply as there is a clear link between mitochondrial structure, specifically supercomplex formation, and mitochondrial bioenergetics (Baker et al., 2019). As such, the reduced CL remodeling due to the knockdown of Taz influences mitochondrial form which may result in impaired mitochondrial bioenergetics, altering muscle contractile kinetics. In fact, individuals with progressive external ophthalmoplegia, a form of mitochondrial myopathy with reduced ATP production, demonstrate slowed contraction and relaxation kinetics in tibialis anterior muscle compared to healthy controls (Moglia et al., 1995). Given the reduced mitochondrial supercomplex formation seen in the current study combined with the characteristics of impaired mitochondrial bioenergetics seen with Barth syndrome, including the TazKD rodent model (Ghosh et al., 2019; Ren et al., 2019), it stands to reason that this could be a significant contributor to the slowed contractile kinetic phenotype. Future research will examine possible correlations between mitochondrial bioenergetics and the impaired contractile kinetic phenotype in TazKD mice.

An alternative hypothesis that may contribute to the slowing of contractile kinetics in TazKD soleus may be impaired calcium homeostasis. To facilitate the contraction-relaxation cycle of skeletal muscle, calcium moves out and in of the sarcoplasmic reticulum, respectively. To regulate mitochondrial ATP production to match skeletal muscle contractile ATP demands, the increased cytosolic calcium seen during contraction enters mitochondria through the mitochondrial calcium uniporter (MCU) and, in turn, upregulates mitochondrial-dependent ATP supply (Finkel et al., 2015). Previous research has shown that cellular models of Barth syndrome (Taz knockout C_2_C_12_ myoblasts and Barth syndrome patient-derived lymphocytes) have reduced levels of MCU and that CL is required for proper MCU activity (Ghosh et al., 2020). It is plausible that the reduced CL content in TazKD impaired soleus mitochondrial MCU content and function, preventing a match between ATP supply and demand, resulting in slower contraction and should be the focus of the future research.

An important contributor to potential impaired calcium homeostasis, and resulting slow contractile kinetics, specifically ½ RT and −d*P*/d*t*, in TazKD soleus is SERCA. SERCA transports calcium back into the SR post-contraction, and its function is reflected in its activity (total amount of calcium moved into the SR) and efficiency (ATP cost per calcium ion moved; Periasamy and Kalyanasundaram, 2007). Changes to SERCA function could delay calcium uptake and, in turn, muscle relaxation. There are seven known isoforms of SERCA with two found in adult skeletal muscle, SERCA1a in fast twitch fibers and SERCA2a in slow twitch fibers and cardiac tissue (Periasamy and Kalyanasundaram, 2007). Previous research has shown that disruptions to SERCA2a gene expression prolonged soleus relaxation time (Sjaland et al., 2011). In addition, left ventricle of TazKD mice compared to wild type demonstrated no difference in SERCA2a expression but had lower SERCA activity that correlated with greater SERCA2a tyrosine nitration, a marker of oxidative/nitrosative stress (Braun et al., 2019). Thus, changes to SERCA2a function in TazKD soleus, be it nitrosative stress or protein expression, may be a contributing factor to slower relaxation kinetics. Future research should examine SERCA2a protein expression and tyrosine nitration in TazKD soleus.

The force-frequency relationship in soleus of TazKD mice compared to wild-type littermates demonstrated a lower force generated at 80 and 100Hz, which is in agreement with a previous study that showed impaired soleus force production at 100 and 160Hz in 2-month old mice (Soustek et al., 2011). The impaired force in TazKD soleus did not translate into a significant reduction in the frequency required for 50% of maximal force (F_50_). However, it is important to note that in addition to impaired soleus relaxation time highlighted above, knocking out SERCA2a resulted in a 9% reduction in F_50_ (Sjaland et al., 2011). This, once again, points to a potential role of SERCA2a form and function in TazKD contractile kinetics that warrants further investigation.

### The Impaired Contractile Phenotype Seen *in vitro* in TazKD Compared to WT Did Not Translate to Altered Muscle Function *in vivo*

Slowed contractile kinetics in TazKD soleus compared to WT did not translate to changes in grip strength. A lack of change to *in vivo* muscle function is similar to previous research in that locomotor activity in TazKD mice did not differ from WT (Cole et al., 2018; Goncalves et al., 2021). Grip strength is a common non-invasive *in vivo* evaluation of muscle force (Ge et al., 2016; Martinez-Huenchullan et al., 2017). However, despite slower rates of contraction and relaxation in soleus, there were no changes in peak twitch force. In addition, contractile properties of one hindlimb muscle may not be representative of all limb muscles. Skeletal muscles vary in terms of fiber-type composition and, in turn, contractile properties (Schiaffino and Reggiani, 2011). It is currently unknown if the doxycycline-induced knockdown of Taz in other muscles with similar oxidative capacities as soleus, such as red gastrocnemius and vastus intermedius (Bloemberg and Quadrilatero, 2012), would show similar impaired contractile kinetics. Future research should examine whole hindlimb contractile properties (Gerlinger-Romero et al., 2019) in TazKD mice to determine if the impaired contractile kinetics is expressed at the level of the whole hindlimb.

### Supplemental LA Attenuated, to Some Degree, the *in vitro* Impaired Contractile Phenotype in the Soleus of TazKD Mice

Diet supplemented with high LA safflower oil for 8weeks somewhat attenuated, although not statistically significant, the total amount of CL and mitochondrial supercomplex formation, but did not increase CL LA content or reduce the MLCL:CL ratio in TazKD soleus. These findings are somewhat in contrast to previous research where supplemental LA reduced the MLCL:CL ratio in Barth syndrome patient-derived lymphoblast (Xu et al., 2016) and cardiomyocytes (Wang et al., 2014), increased CL content and CL 18:2n6 composition in Barth syndrome patient-derived fibroblasts (Valianpour et al., 2003), and increased cardiac tetralinoleoyl CL (TLCL) in rodent models of heart failure (Chicco et al., 2008; Mulligan et al., 2012; Maekawa et al., 2019) that have impaired CL biosynthetic pathways (Saini-Chohan et al., 2009). It is plausible that dietary supplemental LA may have stimulated mitochondrial biogenesis (discussed more below) resulting in a non-significant increase in total CL content in TazKD soleus. However, in the face of reduced taz, this limited CL remodeling and, in turn, CL 18:2n6 composition. Future research should examine all CL species in TazKD soleus using mass spectrometry technology and their relative response to high dietary LA.

The current study demonstrates that time to peak twitch and, to a lesser degree, half-relaxation time were improved with the high LA safflower oil diet with non-statistically significant increases in CL content, CL LA composition, and mitochondrial supercomplex formation. It may be argued that dietary LA imposed, in part, some biologically significant influence on improving the impaired contractile kinetic phenotype of TazKD soleus by way of improved mitochondrial form (CL content and composition and supercomplex formation) and, in turn, mitochondrial bioenergetic function. However, these findings may not fully address the LA supplemented diet-mediated improved contractile kinetic phenotype in TazKD soleus. A complementary hypothesis may be related to the ability of dietary lipids to influence skeletal muscle metabolism and energy production needed during contraction and relaxation independent of changes to mitochondrial structure. It has been reported that the skeletal muscle of Barth syndrome patients (Cade et al., 2019) and TazKD mice (Powers et al., 2013; Goncalves et al., 2021) demonstrate impaired fat oxidation and reliance on glycolytic pathways that produce lactate that is believed to contribute to exercise intolerance. It has also been shown in TazKD mice that bezafibrate, a pan-PPAR agonist, improved cardiac function by increasing mitochondrial biogenesis (Huang et al., 2017; Schafer et al., 2018) with no improvement in TLCL (Huang et al., 2017), and when combined with endurance training, improves exercise tolerance (Schafer et al., 2018). In skeletal muscle, PPARβ/δ is the dominant isoform and activation by lipophilic ligands, including fatty acids, results in increased mitochondrial biogenesis and β-oxidation (Manickam and Wahli, 2017). It can be hypothesized that increased availability of LA may have increased mitochondrial biogenesis and/or β-oxidative capacity in soleus of TazKD mice through PPARβ/δ activation contributing to an improvement to the impaired contractile kinetic phenotype. Future research should examine the role of PPAR as it relates to LA diet-mediated improvement to muscle contraction in TazKD mice.

### Summary and Conclusion

This was the first study to demonstrate an impaired contractile phenotype *in vitro* in soleus of the rodent model of Barth syndrome. However, when this model was presented with supplemental LA in the diet, the improvement in the impaired contractile kinetics appears to be somewhat mediated by mitochondrial structural changes due to non-statistically significant increases in CL content and mitochondrial supercomplex formation post-dietary treatment. A complementary hypothesis is that the improved *in vitro* contractile kinetics may also be through a PPAR pathway, potentially improving lipid metabolism to provide energy for contraction and relaxation. Future research should further examine alternative mechanisms of dietary supplemental LA on improving skeletal muscle contractile dysfunction in TazKD mice.

## Data Availability Statement

The original contributions presented in the study are included in the article/supplementary material, and further inquiries can be directed to the corresponding author.

## Ethics Statement

The animal study was reviewed and approved by the Animal Care and Utilization Committee at Brock University.

## Author Contributions

ME performed the *in vivo* experiments, analyzed the data, and edited the manuscript. DV, MA, MF, and RM assisted in data collection and analyses, and edited the manuscript. SC provided reagents, guidance, and edited the manuscript. PL developed the overall study, collected and analyzed the data, and wrote and edited the manuscript. All authors contributed to the article and approved the submitted version.

## Conflict of Interest

The authors declare that the research was conducted in the absence of any commercial or financial relationships that could be construed as a potential conflict of interest.

## Publisher’s Note

All claims expressed in this article are solely those of the authors and do not necessarily represent those of their affiliated organizations, or those of the publisher, the editors and the reviewers. Any product that may be evaluated in this article, or claim that may be made by its manufacturer, is not guaranteed or endorsed by the publisher.
